# Treatment of Hyperlactatemia in Acute Circulatory Failure Based on CO_2_-O_2_-Derived Indices: Study Protocol for a Prospective, Multicentric, Single, Blind, Randomized, Superiority Study (The LACTEL Study)

**DOI:** 10.3389/fcvm.2022.898406

**Published:** 2022-06-23

**Authors:** Vincenza Caruso, Guillaume Besch, Maxime Nguyen, Sebastien Pili-Floury, Belaid Bouhemad, Pierre-Grégoire Guinot, the Lactel Study Group, Audrey Martin

**Affiliations:** ^1^Anaesthesiology and Critical Care Department, Dijon Bourgogne University Hospital, Dijon, France; ^2^University of Burgundy Franche-Comté, LNC UMR1231, Dijon, France; ^3^Department of Anesthesiology and Intensive Care Medicine, University Hospital of Besançon, Besançon, France; ^4^EA3920, University of Franche-Comté, Besançon, France

**Keywords:** acute circulatory failure, hyperlactatemia, pCO_2_ gap, resuscitation, anaerobic metabolism, sepsis, cardiac surgery shock, lactic acidosis

## Abstract

**Background:**

Hyperlactatemia is a biological marker of tissue hypoperfusion with well-known diagnostic, prognostic, and therapeutic implications in shock states. In daily clinical practice, it is difficult to find out the exact mechanism underlying hyperlactatemia. Central venous to arterial CO_2_ difference (pCO_2_ gap) is a better parameter of tissue hypoperfusion than the usual ones (clinical examination and mixed venous saturation). Furthermore, the ratio between the pCO_2_ gap and p(v–a)CO_2_/C(a–v)O_2_ may be a promising indicator of anaerobic metabolism, allowing for the identification of different causes of tissue hypoxia and hyperlactatemia. The main aim of the study is to demonstrate that initial hemodynamic resuscitation based on an algorithm integrating the pCO_2_ gap and p(v–a)CO_2_/C(a–v)O_2_ ratio vs. usual clinical practice in acute circulatory failure improves lactate clearance.

**Methods:**

LACTEL is a randomized, prospective, multicentric, controlled study. It compares the treatment of hyperlactatemia using an algorithm based on the pCO_2_ gap and P(v–a)CO_2_/C(a–v)O_2_ ratio vs. usual clinical practice in acute circulatory failure. A total of 90 patients were enrolled in each treatment group. The primary endpoint is the number of patients with a lactate clearance of more than 10% 2 h after inclusion. Lactate levels were monitored during the first 48 h of treatment as hemodynamic parameters, biological markers of organ failure, and 28-day mortality.

**Discussion:**

pCO_2_ derivate indices may be of better interest than routine clinical indices to differentiate causes of hyperlactatemia and diagnose anaerobiosis. LACTEL results will provide clinical insights into the role of these indices in the early hemodynamic management of acute circulatory failure in the ICU.

**Clinical Trial Registration:**

www.clinicaltrials.gov; identifier: NCT05032521.

## Introduction

Acute circulatory failure (i.e., shock) is a very common life-threatening condition among patients admitted to intensive care units (ICUs) (1). Historically, treatment of shock has been based on physical examination (skin mottling, capillary refill time, and urine output) and biological macrohemodynamic parameters (blood pressure, arterial lactate, and mixed venous saturation).

During acute circulatory failure, hyperlactatemia remains a parameter widely used to assess tissue hypoperfusion despite its limitations (2). Hyperlactatemia and the clearance of arterial lactate have been demonstrated to be associated with morbidity and mortality in patients with shock and even during/following surgery (3–6). From a physiological perspective, hyperlactatemia can be the result of increased production (type A), reduced elimination (type B), or both (6, 7). Although identification of the exact cause underlying hyperlactatemia is very important for targeted therapy (fluid resuscitation, inotropic or vasopressor support), in clinical practice, it is very difficult to accurately identify the cause of hyperlactatemia (6).

From work carried out over the last 20 years, the pCO_2_ gap has been increasingly recognized as a reliable tool to evaluate tissue perfusion, because it was suggested as a marker of the relationship between cardiac output (CO) and global metabolic demand (8–11). Later, several studies have evaluated the P(v–a)CO_2_/C(a–v)O_2_ ratio as a parameter that was able to provide additional information about anaerobic metabolism in order to identify the causes of hyperlactatemia and to define shock severity (12–14). Based on these studies, authors have proposed different algorithms based on CO_2_-O_2_-derived indices to treat shock and arterial hyperlactatemia (7, 9).

Our hypothesis is that treatment of hyperlactatemia based on CO_2_-O_2_-derived indices would allow for a faster and higher lactate clearance, thus reducing inappropriate therapy and improving outcomes.

## Materials and Methods

### Study Design

LACTEL (*Traitement de l'hyperLACtaTEmie du patient en insuffisance circuLatoire aigue basée sur l'analyse des indices dérivés du CO2: étude randomisée prospective de supériorité*) is a randomized, prospective, multicentric, single-blind, parallel-group, superiority study. It compares the treatment of hyperlactatemia using an algorithm based on the pCO_2_ gap and the pCO_2_ gap/P(a-v)O_2_ ratio vs. usual clinical practice in patients with shock. The study was approved by an independent ethics committee (*Comité de Protection des Personnes Sud Ouest et Outre-Mer* on 8 July 2021, 2021-A101451-40) and was registered (ClinicalTrials.gov: NCT05032521). The study is conducted in ICUs of the University-affiliated tertiary Hospital of Dijon and the University-affiliated tertiary Hospital of Besancon, France. All the patients or their next of kin received written information about the study and gave consent to participate. The design of the study respects the requirements of the Medical Research Involving Human Subjects Act and the Declaration of Helsinki.

Standard protocol items: the study follows the SPIRIT Recommendations for Interventional Trials, and the SPIRIT protocol chronology is presented in Table 1.

**Table 1:** Protocol timeline.

	**ICU admission**	**Enrolment**	**Follow-up**				**End of study**
	**H0 (Day 0)**	**H0 (Day 0)**	**H2**	**H12 (Day 0)**	**H24 (Day 1)**	**H48 (Day 2)**	**Day 28**
Enrolment validation	X						
Randomization		X					
Clinical examination	X	X	X	X	X	X	X
Standard analysis set	X	X	X	X	X	X	
Study analysis set	X	X	X	X	X	X	
Clinical and biological data		X	X	X	X	X	X
Hemodynamic monitoring		X	X	X	X	X	
SOFA score		X	X	X	X	X	X

*ICU, intensive care unit; H, hours at or after enrollment; D, day*.

The first patient was included in December 2021, and the last follow-up was planned until October 2024.

### Study Population

Adult patients are considered for enrollment if they have acute circulatory failure and arterial blood lactate levels ≥3 mmol L^−1^. Acute circulatory failure was defined as systolic blood pressure < 90 mmHg, and/or mean blood pressure <65 mmHg, need for infusion of vasopressor catecholamines, and/or skin mottling, and/or diuresis <0.5 ml kg^−1^ h^−1^ (1, 15). All types of shock will be included: septic, cardiogenic, hemorrhagic, and post-operative.

The inclusion, non-inclusion, and exclusion criteria are provided in Table 2.

**Table 2:** Inclusion, non-inclusion, and exclusion criteria of the study.

**Inclusion criteria**	**Non-inclusion criteria**
• Age ≥ 18 years	• Age <18 years
• Patient or family agreement	• Pregnant or breastfeeding patient
• Arterial blood lactate levels ≥ 3 mmol l^−1^	• No medical insurance
• Acute circulatory failure	• Patient under court order
	• Patient under legal protection • ECMO or Impella support
	**Exclusion criteria**
	• <48 h ICU Lenght of stay

### Study Intervention

Care of the patients is standardized and follows international guidelines and in-hospital protocols (1, 16–19). Preload dependence is assessed using the passive leg raising maneuver and/or the fluid challenge maneuver. Preload dependence is treated with a fluid bolus (250–500 ml) of Ringer Lactate for over 15 min. Cardiac output is optimized first by optimizing preload, and in the absence of preload dependency, by intravenous administration of dobutamine to obtain a cardiac index (CI) >2.5 L min^−1^ m^−2^. Cardiogenic failure is treated by intravenous administration of dobutamine (2.5–10 μg kg^−1^ min^−1^). Cardiac output can be measured by using echocardiography (Affinity or Epiq Philips™), pulmonary artery catheter, trans-pulmonary thermodilution (PiCCO device, Maquet™), or by Pressure Recording Analytical Method (Mostcare, Vygon™, France) (15). Vasoplegic syndrome is treated by intravenous infusion of norepinephrine (0.01 to 2 μg kg^−1^ min^−1^). Additional use of vasopressin if the norepinephrine dose is over 0.2 μg kg^−1^ min^−1^ is permitted. In accordance with the guidelines, we use a threshold of Hb <70 g/L for transfusion, in the absence of coronary artery disease. In patients with coronary artery disease, the threshold for transfusion is Hb <100 g/L. The mechanical ventilation parameters are adjusted on the basis of arterial blood analysis results. Baseline ventilator settings are as follows: respiratory rate of 10–18 breaths/min, tidal volume of 4–8 ml/kg, positive end-expiratory pressure of 5–10 cmH_2_O, a fraction of inspired oxygen of 0.25–1, targeting proper PaO_2_ of 100–120 mmHg, and PaCO_2_ of 35–45 mmHg. None of the non-hemodynamic therapies will be discontinued, and the drug dose regimen will not be modified. These therapeutic strategies do not differ in the two study groups. In the algorithm group, pCO_2_ is measured by central blood gas analysis.

The study intervention consists of treatment of hyperlactatemia following either an algorithm based on the pCO_2_ gap/P(a–v)O_2_ ratio and pCO_2_ gap or usual clinical practice based on macrohemodynamics, respecting randomization. If hemodynamic deterioration occurs despite hemodynamic optimization, a patient is excluded from the study and is treated following usual clinical protocols.

**Figure 1:**
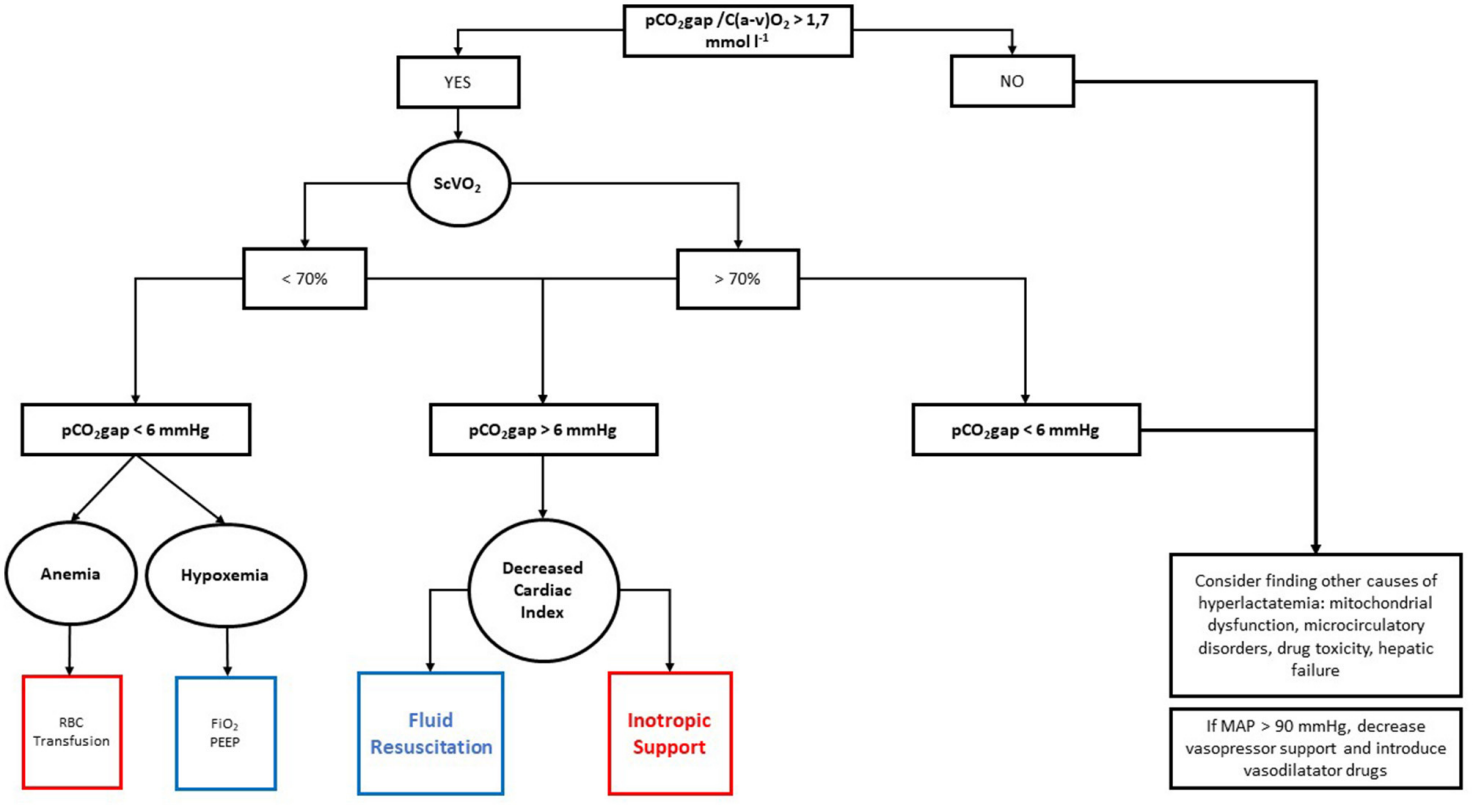
Hemodynamic algorithm based on pCO_2_ gap/P(a–v)O_2_ ratio. RBC, red blood cell; SvO_2_, mixed venous oxygen saturation; PEEP, positive end-expiratory pressure; FiO_2_, fraction of inspired oxygen.

**Figure 2:**
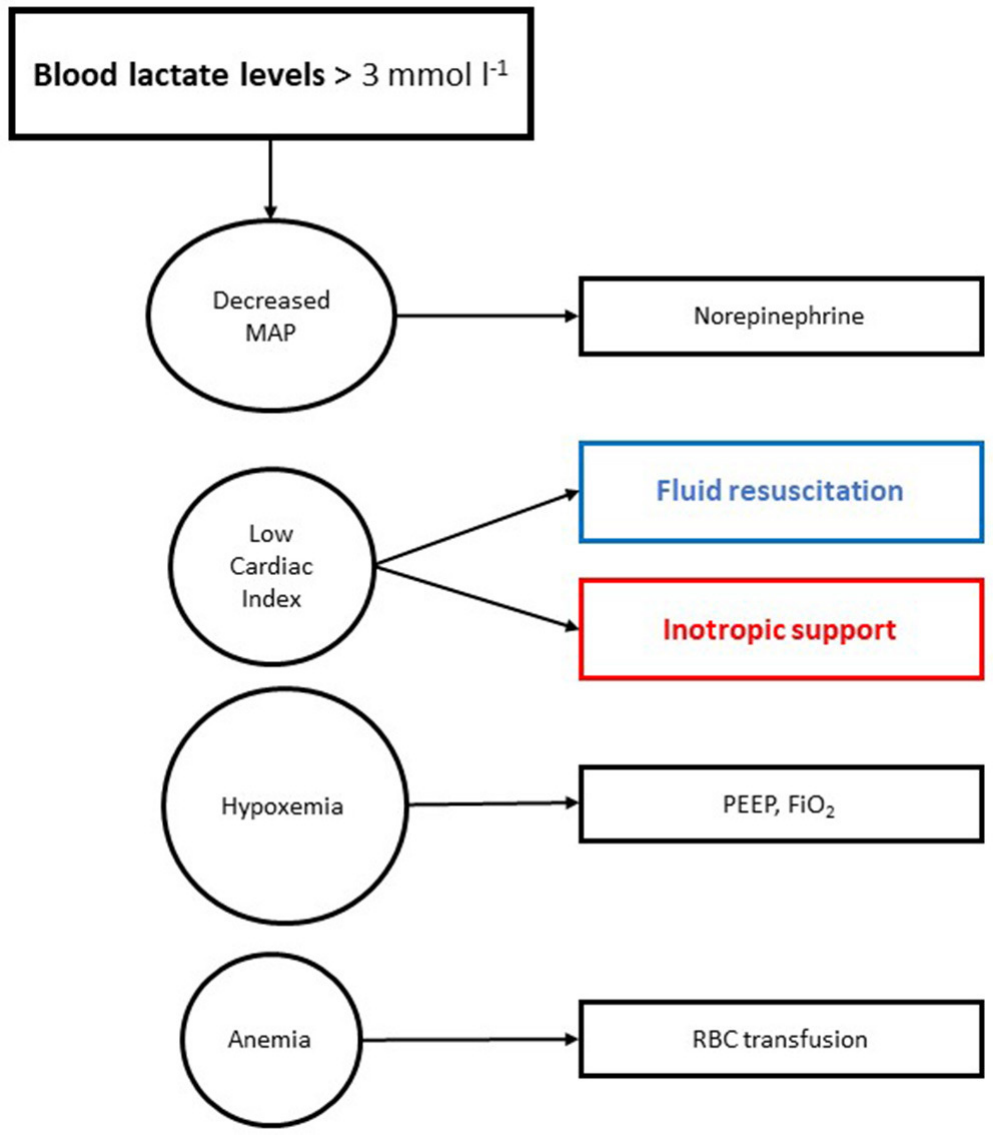
Usual hemodynamic algorithm. MAP, mean arterial pressure; PEEP, positive end-expiratory pressure; FiO_2_, fraction of inspired oxygen; RBC, red blood cell.

The algorithm is shown in Figure 1. Usual clinical practice is shown in Figure 2.

### Randomization

The 1:1 ratio randomization (stratified on center and type of shock) is performed by the investigator after the patient's enrollment, using a dedicated e-platform made with CleanWebTM. The security of this process is assured by individual usernames and newly created passwords for each enrolment. A second check for the inclusion, non-inclusion, and exclusion criteria is required before continuing with the randomization. Technical support is provided by the Research Methodology Support Unit from our university-affiliated hospital, which also keeps a record of the full randomization process. Statistical analysis is performed by a statistician of the Research Methodology Support Unit who is not involved in the study process.

### Objectives

The primary objective is to compare hyperlactatemia treatment effectiveness between an algorithm based on CO_2_-O_2_-derived indices and usual clinical practice after 2 h (3, 20).

The secondary objectives are as follows: (1) to compare lactate clearance at H12 and H24 between the two strategies; (2) to compare the number of patients with blood lactate levels <2 mmol l^−1^ at H12, H24, and H48; (3) to evaluate the ability of P(v–a)CO_2_/C(a–v)O_2_ to predict blood lactate levels > 2 mmol l^−1^; (4) to evaluate the ability of P(v–a)CO_2_/C(a–v)O_2_ to predict lactate clearance after hemodynamic optimization; (5) to evaluate the ability of P(v–a)CO_2_/C(a–v)O_2_ to predict VO_2_ changes after hemodynamic optimization; (6) to compare the number of patients with organ failure; and (7) to compare these effects on ICU length of stay, hospital length of stay, and 28-day mortality.

### Endpoints

The primary endpoint is the number of patients with a lactate clearance over 10% between H0 and H2.

The secondary endpoints are as follows: (1) blood lactate levels at H12, H24, and H48; (2) lactate clearance at H12, H24, and H48; (3) relationship between pCO_2_ gap and blood lactate levels at H2, H12, and H24; (4) relationship between p(v–a)CO_2_/C(a–v)O_2_ and lactate clearance at H2, H12, and H24; (5) relationship between P(v–a)CO_2_ /C(a–v)O_2_ and VO_2_ at H2, H12, and H24; (6) SOFA score on days 0, 1, 2, 7, and 28; and (7) ICU length of stay, hospital length of stay, and 28-day mortality. Patients' follow-up is 28 days after enrolment.

### Data Collection

Data are collected and recorded on eCRF by a physician who is not involved in the care of the patient, and who is blind to the study groups. All patient-related data are anonymously and numerically coded. Data collection encompasses the following: (1) the demographic characteristics of the population (age, sex, weight, SOFA score, SAPS II score, hospital and ICU admission reason, past medical history, and comorbidities), (2) the pharmacological treatment (type and infusion rate of sedation drugs, maximal dose and duration of norepinephrine and dobutamine, amount of fluid, fluid balance, and amount of blood transfusion), (3) the respiratory parameters (tidal volume, respiratory rate, positive end-expiratory pressure, inspiratory O_2_ fraction, end-tidal CO_2_, and plateau and peak pressure), (4) the hemodynamic state (heart rate, arterial blood pressure, pulse pressure, hourly urinary output, cardiac output, respiratory variation of pulse pressure, and/or stroke volume), and (5) the biological marker (hemoglobin, arterial and mixed venous oxygen saturation, partial pressure of CO_2_ in arterial and venous blood, blood lactate level, bicarbonate, base excess, creatinine, sodium, potassium, and chloride).

The study may be discontinued for an individual patient if the patient (or the person designated by the patient) so desires or if decided by the investigator. Every effort will be made to comply with the study protocol. However, the clinician in charge of the patient may deviate from these instructions at any time if he/she considers it necessary. He/she must note this decision and the reason for it in the case report form. If the study is discontinued, no provision is made for patient replacement. Likewise, patients who discontinued the study for whatever reason will receive standard care for the disease in question.

## Statistics

### Sample Size Calculation

Based on actual literature, lactate clearance over 10% over a 2-h period is between 50 and 60%. Assuming our hypothesis that an algorithm based on CO_2_-O_2_-derived indices can reach a 2-h lactate clearance over 80%, 82 patients should be randomized in each treatment group with a bilateral alpha risk of 5% and a power of 80%. After taking into account the risk of missing data and patients lost to follow-up (10% of incomplete data), we fixed the sample size to 180 patients, with 90 in each treatment arm.

### Data Analysis Plan

Normality will be checked graphically with histograms and QQ plots and by the Shapiro-Wilk normality test. Continuous variables will be described as mean ± SD or as median (25–75%, IQR) as appropriate, and categorical variables will be described using a frequency table with a 95% confidence interval for the proportion. The data will be analyzed by intention to treat and per protocol. The two groups will be presented comparatively after randomization. Evaluation of the primary endpoint will be performed by Fisher's exact test. Categorical variables will be compared by Fisher's exact test. Continuous variables will be compared by Student's *t*-test or the Wilcoxon*-*Mann*-*Whitney test, as appropriate. Statistical analysis will be carried out using the R studio software. A *p*-value <0.05 will be considered statistically significant.

## Discussion

Early hemodynamic management of patients with shock is a daily challenge for intensivists. The primary endpoint of each treatment is to correct tissue perfusion by optimizing hemodynamics in order to improve oxygen delivery to tissues (DO_2_), thus meeting tissue oxygen consumption (VO_2_) demands. Macrohemodynamic parameters (i.e., blood pressure and blood lactate levels) have limited adequacy to detect tissue hypoxia because they are often influenced by other factors not strictly linked to circulatory failure. Above all, although the relationship between hyperlactatemia and severity of shock, morbidity, and mortality is well-established (20, 21), high blood lactate levels may be the result of many pathological mechanisms, thus bringing false-positive results and overtreatment.

Over the past 20 years, researchers have constantly been searching for simple indicators that reflect tissue hypoperfusion with accuracy in various settings. The ideal marker should (1) be easy to measure and monitor; (2) should not require technically demanding tests; (3) if needed, the test must be rapid and rapidly available to deliver rapid results, reflecting the current (and not the past) condition of the patient; (4) should maintain its relationship with lactate clearance during ICU stays; and (5) should identify and distinguish anaerobic metabolism from other underlying conditions. In this context, CO_2_-O_2_-derived indices seem to be promising indicators to guide early resuscitation (7, 9).

In particular, the pCO_2_ gap, the difference between venous and arterial pCO_2_, was demonstrated to be associated with mortality in several diseases (sepsis and cardiogenic or perioperative shock) (8, 22, 23). Based on physiological assumptions and simplifications, the pCO_2_ gap was demonstrated to be associated with cardiac output and tissue perfusion (16, 23). In the same way, after studying the pCO_2_ gap, authors have studied the P(v–a)CO_2_/C(a–v)O_2_ ratio (12, 22, 24, 25). This ratio was studied as an indirect blood indicator of respiratory quotient that can detect anaerobic metabolism with better precision than other usual markers. P(v–a)CO_2_/C(a–v)O_2_ ratio may be able to predict VO_2_ responsiveness and hyperlactatemia (7, 14).

Considering the promising evidence, several authors have proposed clinical practice algorithms based on CO_2_-O_2_-derived indices, but the literature on the subject is based on observational studies, and some results appear contradictory (9, 20, 24). The originality of our research is that it consists of a randomized study, allowing us to evaluate the real ability of CO_2_-O_2_-derived indices in the treatment of hyperlactatemia based on the determination of its cause. We focused on arterial lactate clearance during the first 2 h of hemodynamic optimization, because this endpoint was widely studied and demonstrated as associated with mortality (3, 20, 21).

We included the possibility of exclusion of patients in case of hemodynamic degradation. This point can represent a limitation of the study that can decrease the application of the algorithm in a restricted population. Nevertheless, patient care and safety must be taken as a priority. Furthermore, by focusing on the first 2 h of treatment, the results of our study will clarify whether an algorithm based on CO_2_-O_2_-derived indices are able to identify the primary cause of hyperlactatemia, thus providing a better targeted hemodynamic treatment and reducing inappropriate management. If the results confirm our hypothesis, they will represent the first step for further clinical studies. A limitation could be the fact that we did not standardize CO measurement because all CO devices are not interchangeable. However, the same CO technique measure will be used for each patient.

In conclusion, the results of this study will be of major importance in the early treatment of hyperlactatemia during acute circulatory failure and will add further information on the potential clinical use of CO_2_-O_2_-derived parameters in acute care.

## Ethics Statement

This study involving human participants was reviewed and approved by the *Comité de Protection des Personnes Sud Ouest et Outre-Mer* on 8 July 2021. The patients/participants provided their written informed consent to participate in this study.

## Lactel Study Group

Audrey Martin, Mohamed Radhouani, Tiberiu Constandache, Sandrine Grosjean, Pierre Voizeux, Valerian Duclos, Vivien Berthoud, Alexandra Spitz, Marc Ginet, Yannick Brunin, Dejan Ilic, Corentin Evezard, and Laurent Carteron.

## Author Contributions

P-GG, MN, and BB contributed to the conception and design. P-GG, MN, VC, and GB searched for the associated data. VC and P-GG drafted the manuscript. BB provided supervision support. MN performed data analysis. All authors contributed to the critical revision and final approval of the manuscript.

## Funding

The authors perform this study in the course of their normal duties as full-time employees of public healthcare institutions.

## Conflict of Interest

The authors declare that the research was conducted in the absence of any commercial or financial relationships that could be construed as a potential conflict of interest.

## Publisher's Note

All claims expressed in this article are solely those of the authors and do not necessarily represent those of their affiliated organizations, or those of the publisher, the editors and the reviewers. Any product that may be evaluated in this article, or claim that may be made by its manufacturer, is not guaranteed or endorsed by the publisher.
